# Etiological Value of Sterile Inflammation in Preeclampsia: Is It a Non-Infectious Pregnancy Complication?

**DOI:** 10.3389/fcimb.2021.694298

**Published:** 2021-08-16

**Authors:** Sayani Banerjee, Zheping Huang, Zhengke Wang, Akitoshi Nakashima, Shigeru Saito, Surendra Sharma, Shibin Cheng

**Affiliations:** ^1^Department of Pediatrics, Women and Infants Hospital-Warren Alpert Medical School of Brown University, Providence, RI, United States; ^2^Department of Obstetrics and Gynecology, University of Toyama, Toyama, Japan

**Keywords:** preeclampsia, autophagy, endoplasmic reticulum stress, inflammasome, pyroptosis, placenta

## Abstract

Understanding of sterile inflammation and its associated biological triggers and diseases is still at the elementary stage. This becomes more warranted in cases where infections are not associated with the pathology. Detrimental effects of bacterial and viral infections on the immune responses at the maternal-fetal interface as well as pregnancy outcomes have been well documented. However, an infection-induced etiology is not thought to be a major contributing component to severe pregnancy complications such as preeclampsia (PE) and gestational diabetes. How is then an inflammatory signal thought to be associated with these pregnancy complications? It is not clear what type of inflammation is involved in the onset of PE-like features. We opine that sterile inflammation regulated by the inflammasome-gasdermins-caspase-1 axis is a contributory factor to the onset of PE. We hypothesize that increased production and release of damage-associated molecular patterns (DAMPs) or Alarmins such as high-mobility group box1 (HMGB1), cell-free fetal DNA, uric acid, the NOD-like receptor pyrin-containing receptor 3 (NLRP3) inflammasome, IL-1β and IL-18 occur in the PE placenta. Some of these molecules have already been observed in the placenta from women with PE. Mechanistically, emerging evidence has demonstrated that excessive placental endoplasmic reticulum (ER) stress, impaired autophagy and gasdermine D (GSDMD)-mediated intrinsic pyroptosis are key events that contribute to systemic sterile inflammation in patients with PE, especially early-onset PE (e-PE). In this review, we highlight the advances on the roles of sterile inflammation and inflammatory signaling cascades involving ER stress, autophagy deficiency and pyroptosis in PE pathophysiology. Deciphering the mechanisms underlying these inflammatory pathways may provide potential diagnostic biomarkers and facilitate the development of therapeutic strategies to treat this devastating disease.

## Introduction

Along with the two-stage (placental dysfunction and systemic PE pathology) model for preeclampsia (PE), placenta-specific inflammation is also a major component of the responses that lead to the onset of early- or late-onset PE (Redman et al., 1999; Redman and Sargent, 2010; Girard et al., 2014; Harmon et al., 2016; Redman et al., 2020). Although it is not yet well understood what triggers the inflammatory responses in PE, the inflammatory mode does not appear to be caused by infectious agents as the immune responses do not necessarily resemble those elicited by bacterial and viral infections (Girard et al., 2014). A similar case could be made for another late pregnancy malady, gestational diabetes mellitus (Homayouni et al., 2020; Paolino et al., 2021). It is well accepted that elevated inflammation is an important contributor to multiple adverse pregnancy outcomes and potentially to susceptibility to chronic diseases (Cheng and Sharma, 2016a; Kalagiri et al., 2016; Nadeau-Vallée et al., 2016; Ushida et al., 2021). Several pathways could lead to elevated local (maternal-fetal interface) and systemic inflammation that is not caused by infectious agents (Girard et al., 2014; Brien et al., 2019). Although maternal dietary/metabolic patterns, epigenetic modifications, placental stress, intrinsic production of damage-associated molecular patterns (DAMPs), proteinopathy (refers to a group of diseases involving malformed proteins such as protein misfolding and aggregation) and senescence have been described as the effectors of elevated inflammation in PE (Burton and Yung, 2011; Cheng et al., 2016b; Homayouni et al., 2020; Paolino et al., 2021), understanding of these pathways requires further investigation. Most importantly, how these pathways converge on the unscheduled production of DAMPs, autophagy dysregulation, necrosis, trophoblast stress and ensuing defects in their proliferation and differentiation, and placental and systemic proteinopathy is not yet well understood. In this review, we focus on sterile inflammation and its convergence with impaired autophagy, proteinopathy, and metabolic changes in PE.

## Pathogenesis of PE

PE is a multi-factorial and multi-organ syndrome diagnosed by new-onset hypertension and proteinuria at or after 20-week of gestation with subtypes of early- (< 34 gestational weeks) and late- onset (≥34 gestational weeks) (Roberts and Redman, 1993; Redman and Sargent, 2005; Sibai et al., 2005; Saade, 2009; Steegers et al., 2010; Staff et al., 2013; Saito and Nakashima, 2014; Cheng et al., 2016b; Cheng and Sharma, 2016a; Cheng et al., 2019; Nakashima et al., 2019a; Nakashima et al., 2020). As a leading cause of maternal and neonatal morbidity and mortality, PE affects 5-8% of all pregnancies worldwide. Although PE has been recognized as a pregnancy-specific syndrome for over a century, its pathogenesis remains enigmatic (Cheng et al., 2016b). Numerous studies have demonstrated a central role of the placenta in the pathogenesis of PE (Cheng et al., 2016b). This is supported by clinical evidence that PE can occur in a patient with molar pregnancy, and preeclamptic symptoms are mostly normalized right after delivery (Roberts and Redman, 1993; Sibai et al., 2005; Saade, 2009; Steegers et al., 2010; Staff et al., 2013; Cheng et al., 2016b; Nakashima et al., 2020). Inadequate trophoblast invasion, impaired spiral artery remodeling and poor placentation leading to placental hypoxia/ischemia in the placenta have been proposed to be part of the pathological paradigm in PE (Redman and Sargent, 2005; Redman and Sargent, 2010; Burton and Yung, 2011; Lai et al., 2011; Cheng et al., 2016b; Redman et al., 2020). Placental hypoxia/ischemia may induce endoplasmic reticulum (ER) stress, oxidative stress, impaired autophagy, protein aggregate accumulation and cell death such as apoptosis and pyroptosis (Redman et al., 1999; Roberts and Hubel, 1999; Levine et al., 2004a; Burton and Yung, 2011; Cheng et al., 2019; Nakashima et al., 2020). In addition, the ischemic placenta increasingly produces and releases a variety of toxic factors, such as anti-angiogenic factors (i.e. soluble fms-like tyrosine kinase-1 (sFlt-1) and soluble endoglin), syncytial debris, microparticles, pro-inflammatory cytokines and alarmins or damage-associated molecular patterns (DAMPs) (Huppertz et al., 2003; Soleymanlou et al., 2005; Venkatesha et al., 2006; Steegers et al., 2010; Lai et al., 2011; Kalkunte et al., 2013; Cheng et al., 2018). These factors can serve as strong danger signals that trigger inflammation in the absence of microorganisms, a concept known as sterile inflammation or non-infectious inflammation (Matzinger, 2002). Dysregulated sterile inflammation has been linked to a broad range of diseases, including gout, diabetes, neurodegenerative diseases, preterm birth and PE (Romero et al., 2006; Combs et al., 2014; Romero et al., 2014; Cheng and Sharma, 2016a; Kohli et al., 2016; Weel et al., 2017; Liu et al., 2019; Voet et al., 2019; Pirzada et al., 2020). Importantly, epidemiological evidence and experimental studies have demonstrated that enhanced placental sterile inflammation can increase the likelihood of a variety of disorders in mothers and their offspring later in life, such as chronic hypertension, diabetes mellitus, cardiovascular disease, renal disease, hypothyroidism, and thromboembolism, neurodegenerative diseases as well as psychiatric diseases such as autism (Sattar et al., 2003; Bellamy et al., 2007; Vikse et al., 2008; Kvehaugen et al., 2011; Staff et al., 2013; Cheng and Sharma, 2016a).

**Figure 1:**
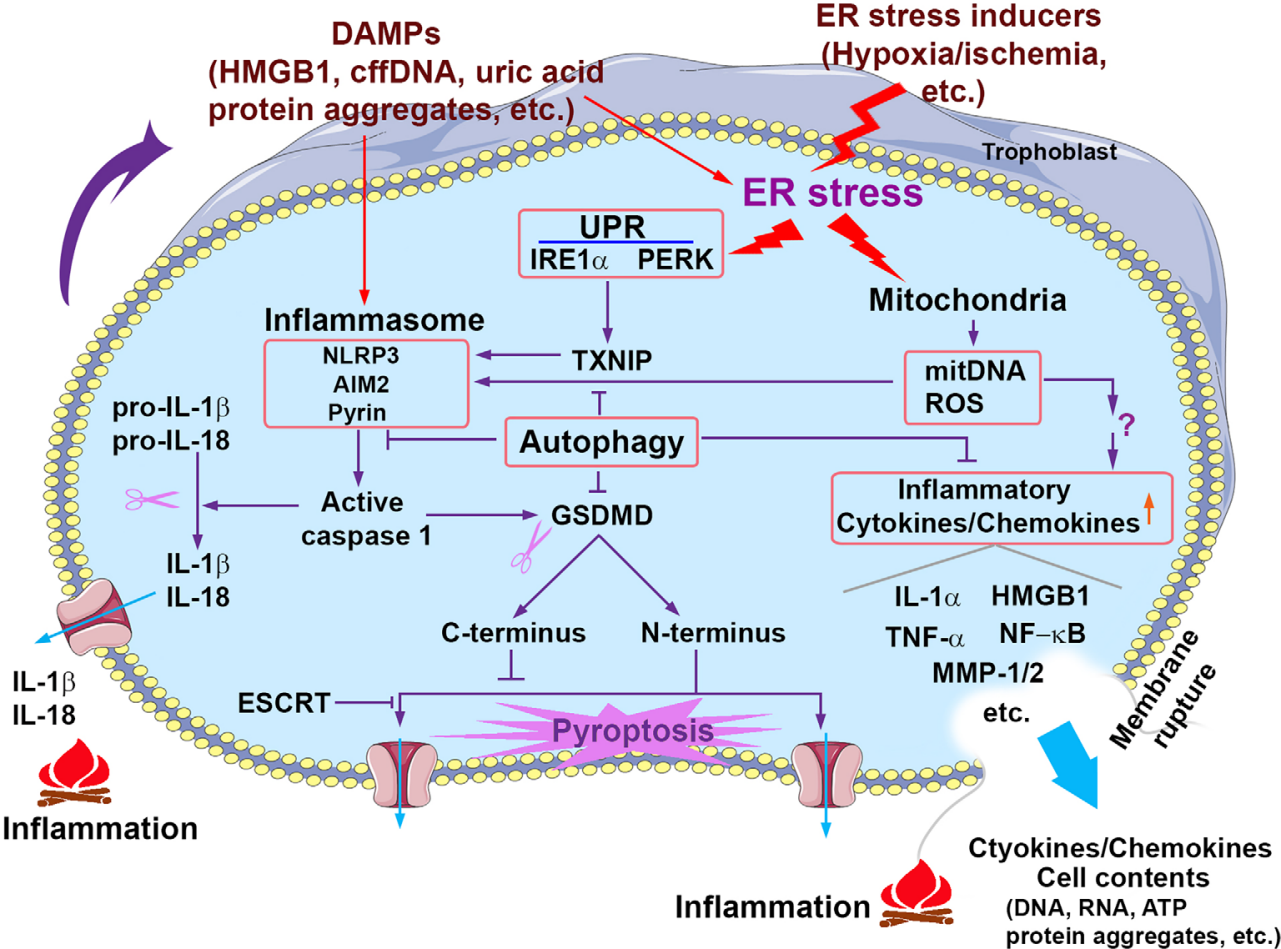
Signaling pathways that contribute to sterile inflammation associated with PE. Prolonged pathological stimuli such as hypoxia/ischemia induce excessive ER stress and the UPR activity and damage the mitochondria, which in turn trigger multiple inflammatory cascades including the activation of inflammasomes, release of mitochondrial DNA (mitDNA) and ROS and consequent production of proinflammatory cytokines/chemokines. Activated inflammasomes can activate caspase 1, which in turn cleaves pro-IL-1β/pro-IL-18 and GSDMD, generating mature IL-1β/IL-18 and GSDMD N-terminus and C-terminus, respectively. GSDMD N-terminus translocates to the plasma membrane and forms the pore structures, which allows the release of proteins and cytokines and may result in pyroptosis. GSDMD C-terminus can serve as an auto-inhibitory regulator for GSDMD N-terminus pore formation. ESCRT (the endosomal sorting complexes required for transport) machinery can repair GSDMD pores-induced membrane damages. Pyroptosis can cause membrane rupture and consequent release of cytokines/chemokines and cell contents. Extracellular cell contents and cytokines/chemokines can activate inflammasomes and induce ER stress of neighbor cells. Autophagy suppresses inflammation through multiple pathways such as inhibiting inflammasome activation and cytokine/chemokine production and release.

As introduced above, we and others have contributed to the evidence that ER stress, oxidative stress, dysregulated autophagy, protein aggregation and cell death occur in the placenta from women with PE (Burton and Yung, 2011; Kalkunte et al., 2013; Buhimschi et al., 2014; Cheng et al., 2019). Notably, *in vitro* experiments can recapitulate these pathological processes in trophoblasts treated with hypoxia or chemical inducers of ER stress (Cheng et al., 2019). It has been shown that ER stress and oxidative stress can remarkably increase the release of an array of cytokines and pro-inflammatory factors from the trophoblast of term villous explants (Cindrova-Davies, 2009; Burton and Yung, 2011; Baker et al., 2021). A new line of investigation on proteinopathy has led to intriguing findings. We and others have shown that protein aggregation is associated with the pathogenesis of PE. Aggregated proteins such as transthyretin and amyloid-β peptides have been observed in the trophoblast layer of the placenta from PE patients and in a cellular model mimicking PE pathophysiology (Kalkunte et al., 2013; Buhimschi et al., 2014; Cheng and Sharma, 2016a; Nakashima et al., 2020). Our recent studies further demonstrated that impaired autophagy may contribute to the accumulation of aggregated proteins (Nakashima et al., 2020). The aggregate complexes involving proteins such as transthyretin can be released into maternal circulation as a cargo of exosomes (Tong et al., 2017) or as large complexes and may function as DAMPs, triggering inflammatory responses. Taken together, ER stress, autophagy, proteinopathy, DAMPs and inflammation are most likely intertwined, synergistically contributing to the multifactorial etiology of PE (**Figure 1**). The following sections focus on the association of these major pathological processes with inflammation and their roles in the pathogenesis of PE.

## DAMPs: The Effectors of Sterile Inflammation in PE

Pregnancy is a unique and well-choreographed physiological process that involves dynamic crosstalk between the maternal immune system and the semi-allogenic placenta (Sharma et al., 2010; PrabhuDas et al., 2015). Uterine tissue damage during implantation and trophoblast invasion and interaction with local immune cells at the maternal-fetal interface lead to the release of a wide range of cytokines, hormones, protein factors, and DAMPs from both fetal tissues and maternal immune cells (Urban et al., 2001; Erlebacher, 2013; Nadeau-Vallée et al., 2016; Shirasuna et al., 2016; Brien et al., 2019). These factors can synergistically contribute to sterile inflammation in the placental microenvironment. Can sterile inflammation at the maternal-fetal interface ignite local and systemic deleterious responses and induce adverse pregnancy complications such as PE? In fact, in normal pregnancy, this sterile inflammatory condition is tightly modulated by the maternal immune system (Nadeau-Vallée et al., 2016). A body of evidence has shown that timely scheduled inflammation plays an essential role in the physiological regulation of normal implantation, decidualization, fetal development and parturition (Mandal et al., 2013). However, heightened sterile inflammation in the fetoplacental unit can trigger maternal systemic inflammation and endothelial dysfunction, contributing to the pathophysiology of PE (Kohli and Isermann, 2017).

The well-known DAMPs are an array of intracellular proteins, peptides, lipids or lipid metabolites and nucleic acids, including high-mobility group box1 (HMGB1), IL-1α, adenosine triphosphate (ATP), uric acid, heat shock protein (HSP) 70, S100 family of proteins, amyloid A, histones, and cell-free fetal DNA (cffDNA) (Bianchi, 2007). In response to cellular damage, stress, or necrotic cell death, DAMPs are released into an extracellular environment or maternal circulation. Similar to infectious inflammatory stimuli, DAMPs can activate pattern recognition receptors (PRRs) such as TLRs and NOD-like receptors, leading to the recruitment of neutrophils and macrophages, the production and release of inflammatory chemokines and cytokines, and the induction of T-cell mediated adaptive immune responses (Wyczanska and Lange-Sperandio, 2020). Intriguingly, we recently demonstrated that protein aggregates might be released from stressed trophoblasts, serving as DAMPs to induce sterile inflammation associated with PE (Tong et al., 2017; Nakashima et al., 2020). Increased levels of DAMPs and their receptors such as TLR 4 have been detected in the PE placenta (Abrahams et al., 2005). Recently, extracellular vesicles have been recognized as an important carrier of protein aggregates, miRNA, mRNA, lipids and apoptotic factors that may mediate inflammatory responses associated with PE pathophysiology (Schuster et al., 2021). Circulating extracellular vesicles exist in greater quantity in the plasma of PE subjects *vs.* normal pregnancy (Gilani et al., 2016; Tong et al., 2017; Schuster et al., 2021; Tong et al., 2021). Below, we will discuss the DAMPs that may be associated with the pathogenesis of PE (**Figure 1**).

### HMGB1

HMGB1 is a highly conserved, chromatin protein for DNA replication, transcription and repair, and nucleosome stabilization (Boonyaratanakornkit et al., 1998; Stros, 2010; Celona et al., 2011). At the same time, modified and circulating HMGB1 has been identified in patients with sepsis (Zhou et al., 2018). The subcellular localization of HMGB1 depends on its posttranslational modification. Upon hyperacetylation (Zhou et al., 2018) and glycosylation (Kim et al., 2016), nuclear HMGB1 translocates to the cytosol. After being released from stressed or dead cells, HMGB1 binds TLRs, such as TLR2 and TLR4, or the receptor for advanced glycation end products (RAGE) and triggers the NF-κB signaling pathway to induce p53-dependent cellular senescence (Sims et al., 2010). A higher level of HMGB1 has been observed in sera from PE patients (Zhu et al., 2015). Hypoxia, not normoxia, increases HMGB1 abundance at both mRNA and protein levels as well as HMGB1 secretion from trophoblasts (Jiang et al., 2014). HMGB1 treatment enhances the secretion of proinflammatory IL-6, IL-8 and MCP-1 in first trimester trophoblasts, leading to increased monocyte migration (Shirasuna et al., 2016). Low Molecular Weight Heparin can inhibit HMGB1/RAGE complex formation in physiological explants, and then modulate PE placentae inflammatory response (Zenerino et al., 2017). Intriguingly, intra-amniotic administration of HMGB1 elicits preterm birth and neonatal death, which can be rescued by treatment with betamethasone (Gomez-Lopez et al., 2016; Galaz et al., 2021)

### Extracellular 70-kDa Heat Shock Proteins (Hsp70)

As molecular chaperones, intracellular Hsp70 protects against DNA damage and protein aggregation and facilitates protein folding, thereby exerting cytoprotective and anti-inflammatory roles. On the other hand, extracellular Hsp70 can act as a DAMP, mediating inflammation (Calderwood et al., 2007). Hsps become pro-inflammatory factors through binding to surface receptors (CD14, CD36, CD40, CD91, LOX-1, TLR2 and TLR4) on antigen-presenting cells, stimulating the release of proinflammatory cytokines (IL-1β, IL-6, and TNF-α) (Molvarec et al., 2010). A prior study has shown that intra-amniotic administration of HSP70 induces significantly increased the rate of neonatal mortality (Schwenkel et al., 2020). Serum Hsp70 protein is elevated in women with transient hypertension of pregnancy, PE and superimposed PE (Fukushima et al., 2005). Hypoxia increases the expression of Hsp70 in trophoblast cells (Ishioka et al., 2006). An elevated serum concentration of Hsp70 has been reported in women with PE and HELLP syndrome (hemolysis, elevated liver enzymes, and low platelets) (Molvarec et al., 2011). Increased Hsp70 levels are related to multiple inflammatory markers such as C-reactive proteins and cffDNA in PE patients compared to normal pregnancy, suggesting a link of Hsp70 to systemic inflammation (Molvarec et al., 2011).

### Uric Acid

Uric acid is a metabolic product of purine through degradation by xanthine oxidase. Uric acid is an antioxidant only in the hydrophilic environment at low concentration in blood (Bowman et al., 2010). A high concentration of uric acid forms needle-like shape crystals, which deposit in the joints, causing acute inflammation in gout. Increased uric acid concentrations were first reported in preeclamptic women in the late 1800s. Studies show that elevated serum uric acid is associated with severe PE (Voto et al., 1988; Bainbridge and Roberts, 2008). Under normal circumstances, uric acid scavenges oxidizing agents and quickly recycles back to its anti-oxidant state. Uric acid elevates the abundance of the chemokine monocyte chemoattractant protein-1 (MCP-1) mRNA and protein in vascular smooth muscle cells in a time and dose-dependent manner (Kanellis et al., 2003). Increased concentration of serum uric acid has been found to be positively correlated to the concentration of circulating TNF-α in women with PE (Zhao et al., 2016). Uric acid treatment results in the production of proinflammatory cytokines (IL-1β, IL-6, and interferon γ) and chemokines (MCP-1 and GROa) in trophoblasts (Brien et al., 2017). Administration of uric acid crystals at late gestational days elicits placental inflammation in rats, suggesting an active proinflammatory role for uric acid at the maternal-fetal interface. (Brien et al., 2017)

### cffDNA

cffDNA is a group of small DNA fragments < 313 bp length that are shed from apoptotic and necrotic placental cells and released into maternal circulation (Reddy et al., 2008; Cheng et al., 2018). cffDNA concentration rises progressively during normal pregnancy and peaks at term and then rapidly drops to undetectable levels postpartum (Reddy et al., 2008; Cheng et al., 2018). cffDNA contains unmethylated CpG motifs similar to bacterial and viral DNA, and thus, can interact with TLR9, leading to the activation of NF-kB and upregulation of proinflammatory cytokines and chemokines (Reddy et al., 2008; Cheng et al., 2018). In addition, cffDNA can activate the Stimulator of Interferon Genes, which in turn complexes with TANK binding kinase, leading to phosphorylation of transcription factors IRF3 and NF-κB (Cheng et al., 2018). Through this TLR9-independent pathway, cffDNA triggers the release of type 1 interferons, IFN-α and IFN-β, and other pro-inflammatory mediators. Thus cffDNA serves as a proinflammatory trigger with unique capability to ignite inflammatory cascades. Elevated levels of cffDNA have been observed in pregnancy outcomes such as gestational diabetes (Thurik et al., 2016) and PE (Levine et al., 2004b; Lazar et al., 2009; Hahn et al., 2011; Vlková et al., 2015; Ozeki et al., 2019). cffDNA isolated from the PE placenta induces both production and secretion of IL-6 and IL-8 in trophoblasts compared to controls (Ozeki et al., 2019). Nevertheless, whether cffDNA is a causative factor or just a consequent byproduct and how cffDNA triggers sterile inflammation in PE pathology remain to be elucidated.

### Toxic Protein Aggregates

We have recently demonstrated that PE is a proteinopathy syndrome (Kalkunte et al., 2013; Cheng and Sharma, 2016a; Tong et al., 2017; Nakashima et al., 2019a; Sharma, 2021). Our data suggest that there is an accumulation of toxic protein aggregates in the placenta from PE patients. In our prior studies, we identified transthyretin (TTR) as a key protein that is dysregulated in PE (Kalkunte et al., 2013). Importantly, administration of TTR immunoprecipitated from PE sera induces PE-like features, including IUGR, hypertension, proteinuria, and glomerular endotheliosis in pregnant IL-10^-/-^ mice (Kalkunte et al., 2013). Our recent studies have shown robust accumulation of protein aggregates involving TTR in the placenta from PE deliveries using immunofluorescence colocalization analysis with TTR antibody (Kalkunte et al., 2013). Interestingly, tissue explants isolated from the PE placenta release increased levels of TTR aggregates, which are carried as the cargoes of micro- and nano-vesicles (Tong et al., 2017). Protein aggregates have been linked with induction of ER stress, oxidative stress and inflammation (Fong and Vieira, 2013; Suenaga et al., 2017). These findings suggest that the placenta-derived TTR aggregates may serve as alarmins, triggering sterile inflammation. In support of this, a protein aggregate complex containing TTR, amyloid-β peptide and other proteins has been detected in sera from PE patients using our novel blood test assay (Cheng et al., 2016b). The molecular mechanisms by which protein aggregates activate inflammation remain yet to be characterized.

## Inflammasome: A Contributor to PE Pathology

### Inflammasome During Normal Pregnancy

The innate immune system utilizes a variety of germline-encoded pattern-recognition receptors (PRRs) to detect invariant microbes. The nucleotide-binding oligomerization domain (NOD)-like receptors (NLRs) are part of the intracellular PRRs that recognize microbe-derived pathogen-associated molecular patterns (PAMPs) or host cell-derived danger-associated molecular patterns (DAMPs) (Akira et al., 2006; Shirasuna et al., 2020). NLRs are a family of proteins that contain a central nucleotide-binding and oligomerization (NACHT) domain, which is flanked by C-terminal leucine-rich repeats (LRRs) and N-terminal caspase recruitment (CARD) of pyrin (PYD) domains. NACHT domains include three distinct subfamilies: the NODs, the NLRPs (NLRP1-14) and the IPAF subfamily (including NLRC4 and NAIP) (Akira et al., 2006; Shirasuna et al., 2020). NLRs, as scaffolds, can assemble with adaptor protein ASC (apoptosis-associated speck-like protein containing a CARD) and caspase 1 into multiprotein complexes or platforms called “inflammasomes” (Rathinam et al., 2012; Strowig et al., 2012). Thus, NLRs-dependent inflammasomes are a large family of complexes including NLRP1, NLRP2, NLRP3, NLRP6 and NLRC4 (Guo et al., 2015) The NLRP3 inflammasome is the most extensively characterized inflammasome (Takahashi, 2014; Guo et al., 2015). Inflammasomes can be activated upon exposure to pathogens, PAMPs, DAMPs and environmental irritants. Activated NLRP3 oligomerizes and interacts with ASC, which results in the recruitment and activation of procaspase 1 into active caspase 1. Active caspase 1 cleaves pro-IL-1β, pro-IL-18 as well as gasdermin D (GSDMD), creating mature IL-1β, IL-18 and active N-terminal fragments of GSDMD, respectively (Bergsbaken et al., 2009). GSDMD N-terminal domains can trigger pyroptosis, a proinflammatory cell death, leading to amplification of inflammatory cascades (Shi et al., 2015a; Shi et al., 2015b; Cheng et al., 2019) (**Figure 1**).

In addition to NLRs-dependent inflammasomes, NLRs-independent inflammasomes, such as AIM2 (absent in melanoma 2) and Pyrin inflammasomes have been reported. The AIM2 inflammasome is activated by cytosolic DNA of microbial or host cell-derived danger signals (Rathinam and Fitzgerald, 2016). The Pyrin inflammasome, the newly discovered NLRs-independent inflammasome, can respond to the bacterial modification and inactivation of Rho GTPases and microtubule disruption and other cytoskeletal modification resulting from microbial infection, rather than the pathogens themselves (Schnappauf et al., 2019; Zheng et al., 2020). More recently, bile acid metabolites by gut microbiota have been shown to activate the Pyrin inflammasomes, implying a new mechanism by which gut homeostasis can influence host innate immune responses (Rutsch et al., 2020).

Inflammasomes have been studied mainly in immune cells of the innate immune system, such as macrophages. An increasing body of evidence, however, shows that inflammasomes and their downstream effectors, caspase-1 and IL-1β are expressed by gestational tissues (e.g., the placenta and chorioamniotic membranes) during normal pregnancy and induce the sterile inflammatory cascades during term parturition to eliminate cellular debris and for the reconstruction of uterine epithelium. (Gomez-Lopez et al., 2019a). Since inflammation plays crucial roles in regulating implantation, pregnancy maintenance and parturition, inflammasomes have been proposed to participate in the regulation of these processes. Indeed, the expression of inflammasome components, including NLRP1, NLRP2, NLRP3, NLRC4 and ASC, and their downstream effectors (e.g. IL-1β and IL-18) have been observed in the placenta and chorioamniotic membranes at a moderate level during normal pregnancy and at a higher level during term parturition (Matias et al., 2015; Stødle et al., 2018; Gomez-Lopez et al., 2019b; Panaitescu et al., 2019; Zhu et al., 2020). These results are consistent with alterations in Alarmins/DAMPs during normal pregnancy. This is not surprising because inflammasomes are commonly activated by Alarmins/DAMPs. Collectively, inflammasomes play a physiological role in regulating normal pregnant processes.

### Excessive Inflammasome Activation Linked to PE

Dysregulated inflammasomes are associated with a number of diseases such as autoinflammatory syndromes, autoimmune diseases, metabolic diseases, and neurodegenerative diseases (Voet et al., 2019; Pirzada et al., 2020). Can alteration of inflammasome activation be invoked in the pathological paradigms of PE? As a matter of fact, we and others have recently provided evidence for a link of enhanced inflammasome activation to the pathogenesis of adverse pregnancy outcomes, including preterm birth and PE (Kohli et al., 2016; Weel et al., 2017; Cheng et al., 2019; Murthi et al., 2020). Genetically, NLRP1 L155H and NLRP3 rs10754558 polymorphisms have been identified as risk factors for PE development (Pontillo et al., 2015; Xu et al., 2018). We found significantly increased levels of NLRP3, active caspase 1 and mature IL-1β and IL-18 in the placenta from early-onset PE compared to gestational age-matched normal deliveries and in primary human trophoblasts treated with hypoxia (Cheng et al., 2019). These findings are consistent with those in prior studies on the placenta from women with severe PE using immunostaining and ELISA (Weel et al., 2017; Liu et al., 2019). A higher abundance of NLRP3 has also been observed in peripheral blood mononuclear cells from PE women *vs.* normal pregnant controls (Matias et al., 2015; Socha et al., 2020). Treatment of monocytes from PE women or even from non-pregnant healthy women with monosodium urate (MSU) increases NLRP3, caspase 1, IL-1β and IL-18 abundance. This effect can be abolished by an NLRP3 inhibitor, glibenclamide (Matias et al., 2015). Similarly, MSU increases the expression levels of active caspase1 and IL-1β primary human trophoblasts; however, knockdown of ASC attenuates MSU-induced IL-1β secretion (Mulla et al., 2011). In addition, NLRP3 can be activated by cholesterol crystals. Interestingly, concentrations of MSU and cholesterol are highly elevated in the plasma from PE patients, which are positively correlated with plasma concentrations of high sensitivity C-reactive protein and sFlt-1 (Stødle et al., 2018).

The implication of NLRP3 inflammasome in the pathophysiology of PE has also been appreciated in animal experiments. Knockout of NLRP3 or ASC blocks angiotensin II-induced elevation of systolic blood pressure in a mouse model of PE (Zhou et al., 2007; Shirasuna et al., 2015). Increased NLRP3 inflammasome activity has been reported in a murine model of hypertension (Shirasuna et al., 2015). The NLRP3 inhibitor, MCC950, can attenuate inflammation and hypertension in mice (Krishnan et al., 2019). Kohli and colleagues reported that administration of procoagulant extracellular vesicles (EVs) induces PE-like features in wild-type C57BL/6 mice but not in NLRP3- or Caspase 1-deficient mice (Kohli et al., 2016). EVs injection increases the presence of NLRP3, caspase 1 and IL-1β proteins in the trophoblasts of mouse placenta (Kohli et al., 2016). These findings were then validated in human trophoblast cell lines and placental tissues from PE women and controls (Kohli et al., 2016). This is consistent with our observations on exosome cargo from the PE placenta containing pathologic aggregated proteins (Tong et al., 2017). Furthermore, dimerization of NLRP3 and adaptor protein ASC is present only in the PE placental protein extracts not that derived from control placental samples (Kohli et al., 2016). Altogether, these results point to a central pathogenetic role for placental inflammasomes in the induction of the PE-like phenotype and placental dysfunction. Therefore, targeting NLRP3 inflammasome or its downstream mediators may foster the development of novel anti-inflammatory therapy for the prevention or treatment of PE.

## ER Stress: A Mechanism for Sterile Inflammation in PE

### Excessive ER Stress and Unfolded Protein Response in PE

Protein folding homeostasis is essential for the execution of fundamental cellular functions. The endoplasmic reticulum (ER) is a key organelle for protein folding, assembly, modification and secretion (Schwarz and Blower, 2016). Pathological stimuli can disturb these processes, leading to protein misfolding and aggregation, a condition called ER stress (Cheng et al., 2016b; Schwarz and Blower, 2016). Within the ER, numerous chaperones are responsible for promoting protein folding, sensing protein misfolding and removing aggregated proteins (Rapoport, 2007). The signaling pathways mediated by these chaperones are collectively known as the unfolded protein response (UPR). The key factor for initiation of the UPR is a heat shock protein family chaperone binding immunoglobulin protein (BiP), which is also known as GRP78 and encoded by the heat shock protein family A member 5 gene (Cheng et al., 2016b). Under normal conditions, BiP binds to three ER transmembrane molecules known as the ER stress sensors or UPR initiators, i.e. inositol requiring enzyme 1 (IRE1α/β), protein kinase RNA-like ER kinase (PERK) and activating transcription factor 6 (ATF6a/b). These sensor proteins initiate three separate signaling pathways of the UPR. Upon misfolded protein accumulation, BiP dissociates from the UPR initiating molecules and binds to misfolded proteins, activating these initiators and subsequently triggering downstream UPR signaling. Activated UPR machinery can either inhibit protein translation to reduce protein synthesis load in the ER lumen, promote protein refolding, increase degradation of misfolded proteins and prevent their aggregation and accumulation. Thus, the UPR machinery plays a critical role in restoring ER homeostasis, thereby promoting cell survival. However, prolonged (excessive) ER stress may become pathologic under chronic conditions. Hyper-activated UPR machinery may exhaust its capacity and allow deleterious effects to propel. Paradoxically, hyper-activated UPR will trigger proinflammatory and cell destructive pathways, leading to sterile inflammation and cell death *via* apoptosis or pyroptosis, respectively.

Excessive ER stress and UPR activity have been associated with a wide range of diseases such as neurodegenerative diseases and diabetes (Hotamisligil, 2010). Accumulating evidence for the association of dysregulated ER stress and UPR activity with the pathology of PE, especially early-onset PE, has recently emerged (Burton and Yung, 2011; Cheng et al., 2016b). Reduced placental perfusion and subsequent placental ischemia may lead to depletion of intracellular glucose concentrations, inhibition of normal glycosylation, accumulation of unfolded or misfolded proteins within the ER cisternae, which synergistically provokes the activation of the UPR. Indeed, increased protein and mRNA levels of many components of the UPR have been detected in the placenta from early-onset PE, including ATF6, ATF4, IRE1a, PERK, eIF2α and BiP (Burton and Yung, 2011; Yung et al., 2014; Cheng et al., 2016b). These results suggest activation of the UPR in the placenta from early-onset PE. However, the question arises as to whether placental UPR machinery is hyper-activated or not. In this regard, prior studies show that ER stress-induced upregulation of ATF4 and ATF6 may reduce PIGF transcription, which in turn negatively influences maternal endothelial cell function (Powe et al., 2011). The abundance of CHOP (C/EBP homologous protein) and caspase 12 proteins and their mRNA are increased in the placenta from women with early- and late-onset PE, suggesting an occurrence of apoptosis in the placenta (Fu et al., 2015). Our recent work also demonstrates excessive ER stress and UPR activity in the placenta from early-onset PE as evidenced by augmented contents of BiP, PERK and IRE1α (Cheng et al., 2019). Importantly, we found that thioredoxin-interacting protein (TXNIP), the cell fate switch and gasdermin D, an executioner of pyroptosis are heavily accumulated in the placenta from preeclamptic women, implying that the UPR is hyper-activated (Cheng et al., 2019) (see the section below). Interestingly, these molecular events can be fully recapitulated in a cellular model of PE (Cheng et al., 2019).

### ER Stress-Mediated Inflammatory Pathways in PE

Under chronic hypoxic microenvironment at the maternal-fetal interface, ER-stressed syncytiotrophoblasts over-secret pro-inflammatory cytokines and release dead cell debris that culminates in enhanced inflammation and endothelial cell dysfunction. Further studies have reported that placental ischemia reduces intracellular levels of ATP, compromises the functioning of the BiP chaperone proteins, and influences calcium release from the ER by altering thiol groups on the calcium channel proteins, which impairs redox balance within the cell (Hool and Corry, 2007; Zhang and Kaufman, 2008). Calcium imbalance may further result from the competitive binding of BiP to misfolded proteins because BiP serves to plug unoccupied translocons and prevent calcium leakage from the membrane under normal conditions (Burton and Yung, 2011). Under chronic hypoxic conditions, activated phosphorylated IRE1 binds the adaptor protein TNF receptor activating factor 2 (TRAF2), which activates the JNK/AKT pathway and phosphorylates NF-κB protein IκB kinase, leading to cleavage of IκBα and activation of NF-κB. On the other hand, ER stress-induced autophosphorylation of PERK results in the phosphorylation of eIF2α, which can decrease IκBα production and thereby induce NF-κB transcription. Ultimately, the release of Ca^2+^ from the ER lumen induces the opening of mitochondrial membrane transition pores and subsequent loss of mitochondrial membrane potential, which leads to the generation of the reactive oxygen species (ROS). In addition, the release of UPR-independent Ca^2+^ and ROS into the cytosol has also been proposed to activate NF-κB, inducing inflammatory responses. Whether these inflammatory pathways are implicated in the pathogenesis of PE remains to be explored.

TXNIP has an important role in enhancing inflammation *via* the ASK1-JNK/p38 and TXNIP/thioredoxin/NF-κB pathway (Bi et al., 2016). Recent findings also implicate TXNIP in modulating the innate immune system: TXNIP binds and activates the NLRP3 inflammasome, eventually leading to the maturation of proinflammatory cytokine IL-1β from pro-IL-1β (Zhou et al., 2010; Li et al., 2019). Our recent studies demonstrate that alleviation of ER stress and inhibition of TXNIP attenuate hypoxia-induced increase in NLRP3 (nucleotide-binding oligomerization domain (NOD)-like receptor (NLR) protein 3) inflammasome and cell death in primary human trophoblasts (Cheng et al., 2019) (**Figure 1**). Our findings suggest that ER stress and UPR hyper-activation can induce inflammation through TXNIP and TXNIP may serve as a liaison between ER stress and inflammation (**Figure 1**). Notably, these results are now supported by other publications. (Abais et al., 2014). Nevertheless, whether excessive ER stress and UPR activity can activate other inflammatory signaling pathways and the mechanisms underlying these complicated processes remain to be investigated.

## Pyroptosis: A Novel Sterile Inflammatory Pathway

### Biochemical Characteristics for Pyroptosis

Programmed cell death is a central cellular mechanism for regulating tissue and organ development by maintaining cellular homeostasis to mount a defense against pathogens. Apoptosis is the prototype of programmed cell death that has been most extensively studied and documented (Fink and Cookson, 2005; Kroemer et al., 2009; Shi et al., 2017). During apoptosis, cell debris and contents are packed in apoptotic bodies that can be engulfed by surrounding macrophages, thereby not inducing inflammatory responses (Fink and Cookson, 2005). Research on apoptosis in response to diverse treatments or infections also led to initial confusing conclusions (Shi et al., 2017). Pyroptosis was originally observed in mouse macrophages treated with anthrax lethal toxin or infected with *Shigella flexneri*; however, it was mistakenly described as “apoptosis”, although treated cells exhibited the activation of caspase-1 and release of IL-1β and intracellular contents (Zychlinsky et al., 1992; Hilbi et al., 1998; Brennan and Cookson, 2000). To distinguish it from immunologically silent apoptosis, this cell death mode was first coined as pyroptosis in 2001 to reflect its proinflammatory nature (Cookson and Brennan, 2001).

Morphologically, pyroptotic cells exhibit some common features of cell death such as membrane blebbing, cell shrinkage, DNA fragmentation, and chromosome condensation as observed in apoptosis (Vande Walle and Lamkanfi, 2016). However, pyroptosis has its morphological hallmark, the formation of a pore structure with 15-32 nm of diameter in the plasma membrane (Ding et al., 2016; Liu et al., 2016). These pores allow transmembrane influxes of ions and fluid, leading to cytoplasmic swelling and eventual plasma membrane rupture, cell osmotic lysis, and release of intracellular proinflammatory contents (de Vasconcelos et al., 2019). Evidence shows that proinflammatory cytokines such as IL-1β and IL-18 can be released through the pores in the plasma membrane in response to pyroptosis (Russo et al., 2016; Heilig et al., 2018). These released danger signals can trigger inflammatory responses which have been termed sterile because of their non-association with infectious agents (**Figure 1**).

While pyroptosis has been documented mainly in cells of the myeloid lineage such as dendritic cells, neutrophils, and macrophages (Orning et al., 2019), emerging evidence also shows its occurrence in CD4+ T cells and non-immune cells such as keratinocytes, endothelial and epithelial cells, neurons, and cancer cells in response to pathogens and DAMPs (Broz et al., 2020; Fang et al., 2020; McKenzie et al., 2020). Pyroptosis has been closely associated with a variety of diseases such as atherosclerosis, sepsis, diabetes, neurological diseases and cancers (Broz et al., 2020; Fang et al., 2020; McKenzie et al., 2020). Interestingly, our recent work demonstrates that pyroptosis occurs in primary human trophoblasts exposed to hypoxia or ER stress inducers as well as in the placenta from women with early-onset PE (Cheng et al., 2019). In addition, the association of pyroptosis with other adverse pregnancy outcomes such as preterm birth has been reported (Gomez-Lopez et al., 2019c). Chorioanmiotic membranes and decidual stromal cells isolated from women with preterm birth display higher levels of pyroptosis than controls (Gomez-Lopez et al., 2019c).

### Gasderminization of Pyroptosis

Gasdermins were identified as pore-forming executioners of pyroptosis by two independent groups in 2015 (Kayagaki et al., 2015; Shi et al., 2015a; Shi et al., 2015b). This groundbreaking discovery has significantly enhanced our understanding of the mechanisms underlying pyroptosis. Gasdermins are a large family of conserved proteins, including gasdermin A, B, C, D, E and DFNB59 with GSDMD being the most studied of all the members (Orning et al., 2019; Broz et al., 2020). GSDMD consists of an N-terminal domain, a C-terminal domain and a flexible linker between two domains. Activation of the pore-forming activity of gasdermins requires proteolytic cleavage of GSDMD at position D276 in mice and D275 in humans by inflammatory caspases-1/4/5/11 to generate free cytotoxic N-terminal fragments and remove C-terminal fragments (Orning et al., 2019; Broz et al., 2020). The N-terminal fragment possesses a strong affinity to acidic phospholipids such as phosphoinositides and cardiolipins and only a weak affinity to phosphatidic acid and phosphatidylserine (Ding et al., 2016). These lipophilic features facilitate the binding of gasdermins to the plasma membrane. Indeed, gasdermins can bind to phosphoinositides in the cytoplasmic leaflet of the plasma membrane and then homo-oligomerize, leading to pore formation (Broz et al., 2020). The dynamic process of GSDMD pore formation has been directly visualized by using a high-resolution atomic force microscope; GSDMD N-terminus embed in the lipid membrane and assemble into arc or slit-shaped intermediate oligomers and then form a ring-shaped transmembrane pore structure (Liu et al., 2019). Thus, pyroptosis is also known as gasdermin-mediated programmed necrosis. On the other hand, C-terminal fragments can translocate to N-terminal domains, which masks the lipid-binding moiety in the N-terminal domains and consequently inhibit the ability of the N-terminal fragments to form the pore structures (Liu et al., 2016). It has been observed that the C-terminal domain of all gasdermins except DFNB59 serves as an inhibitory regulator for pyroptosis (Liu et al., 2016).

The pore formation of gasdermins in the plasma membrane generally results in membrane permeabilization and rupture, leading to cell necrotic lysis; however, this is not always the case. Emerging evidence has revealed that gasdermin pores can mediate cell lysis-independent activities (Evavold et al., 2018; Heilig et al., 2018; Lieberman et al., 2019). For instance, the N-acetyl glucosamine fragment of bacterial peptidoglycan elicits a GSDMD-dependent release of mature IL-1β from mouse macrophages without leading to cell lysis (Heilig et al., 2018; Broz et al., 2020). The large size of GSDMD pores has been shown to allow the direct release of inflammatory cytokines, such as mature IL-1β and IL-18, other small cytosolic proteins, such as small GTPases, and galectins, as well as exosomes (Vande Walle and Lamkanfi, 2016; Lieberman et al., 2019; Orning et al., 2019; Broz et al., 2020). In addition, gasdermin pores may function as large, non-selective membrane channels, regulating ion-mediated inflammatory signaling transduction (Broz et al., 2020). It is considered that the consequences of gasdermin pore formation depend on the levels of gasdermin expression, the amount and size of pores, cell types, the context of different intrinsic and extrinsic signals, and balance with counteracting mechanisms (Broz et al., 2020). As a counteracting mechanism, the endosomal sorting complexes required for transport (ESCRT) machinery can inhibit GSDMD pores-induced pyroptosis by repairing membrane damages caused by GSDMD pores (Rühl et al., 2018) (**Figure 1**). However, the mechanisms by which gasdermins balance between cell lysis-independent signaling pathways (sublytic phase) and cell death-inducing pathways (lytic phase) still remain poorly understood.

The high affinity of gasdermins to membrane lipids raises the question of whether gasdermins can target the membrane of organelles such as the mitochondria and nucleus and then form pores. In this regard, GSDMD N-terminal domains have been shown to bind to cardiolipin in the mitochondrial membrane, which increases the production of reactive oxygen species (Lin et al., 2015; Shi et al., 2015a; Shi et al., 2015b; Broz et al., 2020). Gasdermin A3 N-terminus can translocate to the mitochondria and permeabilize the mitochondrial membrane, leading to mitophagy activation (Lin et al., 2015). Likewise, GSDMD N-terminus binds to the nuclear membrane and disrupts it, resulting in DNA extrusion in neutrophils (Broz et al., 2020). A recent study shows that gasdermin E forms pores in the mitochondrial membrane in cancer cells and concomitantly induces mitochondrial permeabilization and chromosome c release, leading to apoptosis (Rogers et al., 2019). This suggests that gasdermins may function as tumor suppressors. Therefore, gasdermins not only regulate pyroptosis but also mediate other programmed cell death such as apoptosis.

Gasdermins have been widely studied in immune cells with pathogen infection, but little is known about their roles in placental cells especially in the placenta from adverse pregnancy outcomes without pathogenic infection. Our recent studies demonstrate that GSDMD is only moderately expressed in the placenta from normal pregnancy but remarkably elevated in the placenta from women with PE, especially early-onset PE (e-PE) as compared to gestational age-matched controls (Cheng et al., 2019). Notably, immunofluorescent analysis reveals that GSDMD is predominantly localized to the apical surface of syncytiotrophoblasts of microvilli in the e-PE placenta. In contrast, GSDMD immunoreactivity is undetectable in cytotrophoblasts and stromal cells of microvilli (Cheng et al., 2019). Our data suggest the occurrence of increased pyroptosis in syncytiotrophoblasts in the placenta from e-PE patients. Additionally, increased abundance of GSDMD has also been observed in the amniotic fluid, chorioamniotic membranes and decidual stromal cells in women with preterm birth in the context of sterile intro-amniotic inflammation or intra-amniotic infection (Gomez-Lopez et al., 2019c). Whether other gasdermin family members are also expressed in the placenta and involved in physiological regulation in normal pregnancy or pathophysiological processes associated with PE or other pregnancy-related complications remain to be investigated.

### Signaling Pathways Regulating Pyroptosis and Inflammation

Pyroptosis is gasdermin-mediated cell necrotic lysis. Thus, any molecules and signaling pathways that influence gasdermin expression, cleavage and translocation have the ability to modulate pyroptosis and inflammatory responses such as the release of IL-1β and IL-18. Activation of gasdermins is dependent on cleavage by proinflammatory caspases such as caspase 1/4/5/11. There are two distinct pathways that trigger the activation of caspase 1 and caspase 4 in humans or caspase 1 and caspase 11 in mice: the canonical inflammasome and non-canonical inflammasome pathways (Broz et al., 2020). Pathogen-derived or host cell-derived danger signals can trigger pyroptosis through activating a variety of inflammasomes. For instance, NLRP3 inflammasome can be activated by bacteria, viruses, lipopolysaccharides, DAMPs, ROS, extracellular RNA, urate, cholesterol and pore-forming toxins (Takahashi, 2014; Guo et al., 2015; Lebeaupin et al., 2015). AIM2 and pyrin inflammasomes can sense pathogens-derived and endogenous double-stranded DNA and inactivation of host Rho GTPases, respectively (Rathinam and Fitzgerald, 2016). After activation, these inflammasomes bind to the adaptor protein ASC, which activates caspase 1 and consequently leads to pyroptosis. In the non-canonical inflammatory pathway, caspase 4 and caspase 5 in humans (caspase 11 in mice) can be directly activated by cytosolic lipopolysaccharides from invading Gram-negative bacteria or host-derived oxidized phospholipids, which in turn cleave GSDMD and induce pore formation, leading to pyroptosis. In addition, caspase 3 and caspase 8 have been reported to cleave gasdermin E and GSDMD without activation of inflammasomes, respectively (Broz et al., 2020). Although inflammatory signaling pathways regulating pyroptosis have been intensively studied in immune cells with pathogens or sterile infection, little is known about it in the placenta from normal pregnancy and adverse pregnancy complications.

Our recent studies have demonstrated that chronic hypoxia treatment or ER stress inducers increase the expression of NLRP3, active caspase 1, binding immunoglobulin protein (BiP, an ER stress marker), key components of unfolded protein response (UPR) (i.e. inositol-requiring kinase-1 α (IRE1α) and protein kinase R (PKR)-like endoplasmic reticulum kinase (PERK)), as well as TXNIP, a regulatory liaison between ER stress and inflammation, in primary human trophoblasts (Cheng et al., 2019). We further show that blockage of IRE1α or PERK attenuates hypoxia-induced upregulation of TXNIP and caspase 1 cleavage, and inhibition of TXNIP abolishes hypoxia-induced upregulation of NLRP3 and active caspase 1, thereby inhibiting pyroptosis. These findings indicate that excessive ER stress and UPR activity can activate NLRP3 inflammasome and caspase 1, leading to pyroptosis and release of mature IL-1β and IL-18 in human primary trophoblasts exposed to chronic hypoxia, a key pathologic process in the PE etiology (Cheng et al., 2019). Notably, the placenta from patients with e-PE exhibits high levels of BiP, IRE1α, PERK, TXNIP, NLRP3, cleaved caspase 1, cytotoxic N-terminal fragments of GSDMD proteins and proinflammatory cytokines IL-1β and IL-18 compared to gestational age-matched controls (Cheng et al., 2019). Thus, ER stress-NLRP3 inflammasome-pyroptosis-inflammation axis may contribute to the pathophysiology of PE especially e-PE. However, there are many questions remaining to be addressed as to whether other inflammasomes or non-inflammasome molecules are also involved in the activation of pyroptosis and inflammatory responses in the PE pathology. Importantly, how pyroptotic trophoblasts interact with immune cells at the maternal-fetal interface from PE patients and whether dysregulated UPR-induced proteinopathy symptoms (accumulation of protein aggregates) elicit pyroptosis and inflammatory response in the placenta from PE patients are poorly understood.

## Autophagy and Inflammation in PE

### Impaired Autophagy Linked to PE

Autophagy is an evolutionarily conserved cellular process that degrades a bulk of damaged organelles, long-lived proteins, protein aggregates and pathogens through a lysosomal degradation pathway (Huber and Teis, 2016). It includes three distinct pathways, namely macroautophagy, microautophagy and chaperone-mediated autophagy with macroautophagy (or autophagy for short) most widely studied (Oku and Sakai, 2018; Nakashima et al., 2019b; Nakashima et al., 2021). Autophagy including mitophagy can be activated by a variety of stimuli, such as starvation, hypoxia, protein aggregation and infection, and involves a series of dynamic formation of organelles (Ryter et al., 2013). First, isolation membranes form through budding off the ER-mitochondria contact site, elongate, engulf the cargoes, and then close, generating the vacuoles with double membrane called autophagosomes. Autophagosomes then fuse with the lysosomes, forming autolysosomes in which the cargoes are degraded by lysosomal hydrolases. Autophagy plays essential roles in cell survival, bioenergetics, homeostasis, and organism development (Hamasaki et al., 2013).

We have previously discussed that autophagy plays pivotal roles in regulating embryogenesis, trophoblast invasion and vascular remodeling and placentation (Nakashima et al., 2017). Dysregulated autophagy is involved in a large number of diseases such as neurodegenerative diseases and cancers. It is generally accepted that alteration of the autophagy activity is associated with the PE pathology; however, whether the autophagy activity is increased or impaired in the placenta of PE patients has been controversial (Oh et al., 2008; Chen et al., 2012; Akaishi et al., 2014; Saito and Nakashima, 2014; Gao et al., 2015; Melland-Smith et al., 2015; Zhang et al., 2016; Akcora Yildiz et al., 2017; Ozsoy et al., 2018). Most studies mainly focus on the detection of initial stage markers of autophagy pathways, such as LC3-II, Beclin-1, and SQSTM/P62, and suggest increased autophagy activity in the PE placenta. For instance, elevated expression of LC3 and Beclin-1 and reduced abundance of SQSTM1/p62 have been described in the placenta from PE women in comparison to normotensive controls (Oh et al., 2008; Gao et al., 2015; Melland-Smith et al., 2015). On the other hand, emerging evidence has shown the impairment of the autophagy-lysosomal pathway in the PE placenta (Saito and Nakashima, 2014; Ozsoy et al., 2018; Sharma, 2018; Nakashima et al., 2019b; Nakashima et al., 2020).

**Figure 2:**
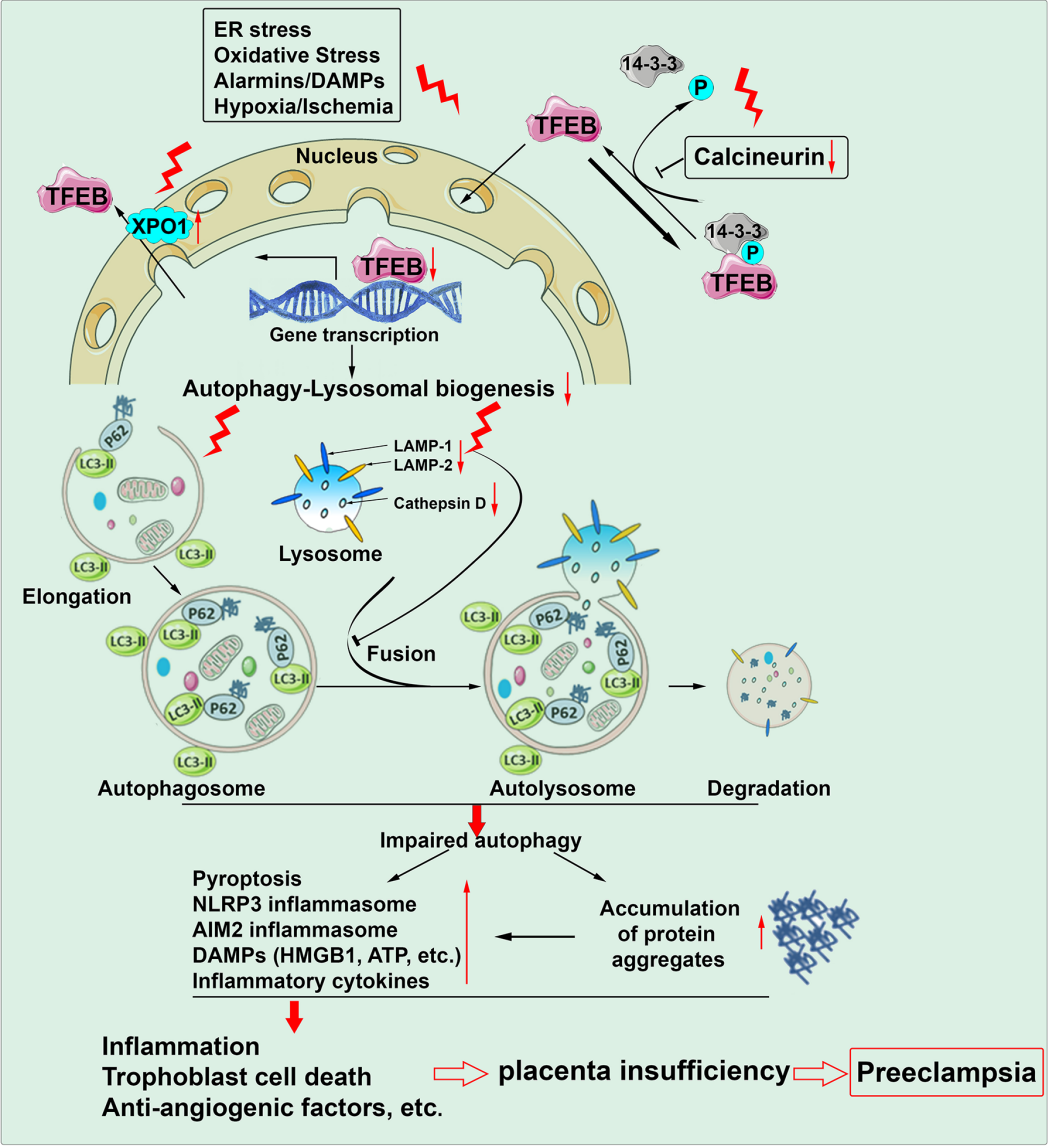
Autophagy machinery is impaired in the placenta of preeclampsia. Chronic stimuli such as ER stress, oxidative stress, alarmins/DAMPs and hypoxia/ischemia can induce downregulation of calcineurin, upregulation of XPO1 (exportin 1) and subsequent inhibition of TFEB nuclear translocation. As a result, autophagy-lysosomal biogenesis is compromised. Impaired autophagy machinery increases the occurrence of pyroptosis, formation of inflammasomes, production of DAMPs/alarmins and inflammatory cytokines and accumulation of protein aggregates. All these pathological paradigms can culminate in sterile inflammation, trophoblast cell death and anti-angiogenic response and consequent placental insufficiency, contributing to the pathogenesis of preeclampsia.

Our recent study provides compelling evidence for impaired autophagy-lysosomal machinery in the PE placenta (Nakashima et al., 2020). Transcription factor EB (TFEB) is a master regulator of lysosomal biogenesis and autophagy activity (Parenti et al., 2015). Upon dephosphorylation by the calcineurin phosphatase, TFEB dissociates with 14-3-3 protein, a cytosolic chaperon protein and translocates to the nucleus, affecting the expression of multiple genes that control the biogenesis of the lysosomes and formation of autophagosomes and autolysosome (fusion with the lysosomes) (Parenti et al., 2015) (**Figure 2**). Our immunofluorescence staining revealed a significant decrease in the content of cytosolic and nuclear TFEB and the expression of LAMP1/2 (lysosome-associated membrane protein 1/2) and cathepsin D in trophoblasts of the placenta from women with severe PE versus normal pregnancy. The PE placenta also exhibited a decreased abundance of calcineurin phosphatase and increased expression of XPO1 (exportin 1) (Medina et al., 2015; Napolitano et al., 2018). XPO1 functions as the nuclear transport receptor for exporting proteins and various RNA from the nucleus to the plasma. Thus, these findings suggest the inhibition of TFEB nuclear translocation in the PE placenta. Since autophagy-lysosomal machinery is responsible for the degradation of protein aggregates, its impairment should culminate in the accumulation of protein aggregates in the PE placenta. To confirm it, we stained the PE placenta using ProteoStat dye and found a robust abundance of protein aggregates located to the trophoblast layer only in the PE placenta, not controls (Nakashima et al., 2020). Interestingly, these molecular events can be fully recapitulated in our cellular model of PE using hypoxia treatment. For example, decreased expression of LAMP1/2, cathepsin D, nuclear TFEB, and calcineurin phosphatase and increased abundance of protein aggregates occur only in hypoxia-treated primary human trophoblasts (Nakashima et al., 2020). Notably, ultrastructural analysis demonstrates that hypoxia remarkably decreases the number of autophagosomes in primary human trophoblasts compared to normoxia using transmission electron microscopy. Taken together, autophagy-lysosomal biogenesis is compromised in the PE placenta, and impaired autophagy machinery may contribute to the accumulation of protein aggregates implicated in PE pathogenesis (Nakashima et al., 2020) (**Figure 2**).

The role of autophagy impairment in PE-related pathophysiology has been evaluated in animal experiments. Trophoblast-specific *atg*7 knockout mice were generated using a lentiviral vector (Aoki et al., 2018; Sharma, 2018). Atg7 is a key component of autophagy flux that regulates autophagosome elongation and conjugation with LC3. Thus, the knockout mice display autophagy deficiency only in the placenta. Placental autophagy deficiency results in shallow trophoblast invasion, failure of vascular remodeling and accumulation of p62 in mouse placenta (Aoki et al., 2018). Using this placental *atg*7 knockout mouse, we further demonstrated reduced TFEB expression and increased accumulation of protein aggregates in the placenta. Most importantly, placental *atg*7 knockout mice exhibit PE-like features such as hypertension (Aoki et al., 2018). These findings suggest a direct causative link between autophagy impairment and PE pathology.

### Autophagy-Mediated Inflammatory Cascades in PE

The autophagy machinery not only degrades damaged organelles and protein aggregates but also sequesters and digests intracellular pathogens, a direct way for autophagy to contribute to immunity. Accumulating evidence has shown critical roles for autophagy in the development and function of immune cells, innate immune signaling and cell-autonomous defense (Matsuzawa-Ishimoto et al., 2018). Autophagy plays a crucial role in inhibiting inflammation through multiple inflammatory pathways. For instance, autophagy can sequester damaged mitochondria that release mitochondrial DNA and ROS and subsequently degrade the key signals for NLRP3 inflammasome activation (Nakahira et al., 2011). Depletion of LC3-II or Beclin-1 promotes the mitochondrial DNA cytosolic translocation and enhances the activation of NLRP3 and caspase-1 inflammasome and the secretion of IL-1β and IL-18 (Nakahira et al., 2011). In addition to NLRP3 inflammasome, autophagy also can suppress the AIM2 inflammasome by directly eliminating inflammasome subunits (Liu et al., 2016). Moreover, autophagy can inhibit the release of DAMPs such as HMGB1 and ATP from dead cells through blocking apoptosis and pyroptosis (Yang et al., 2015). Numerous studies have linked impaired autophagy with a multitude of infectious and inflammatory diseases, such as inflammatory bowel disease, diabetes and obesity (Lapaquette et al., 2015). However, much less has been known about whether autophagy-mediated inflammatory alterations are associated with adverse pregnancy complications, especially PE. In the mouse model of preterm birth, downregulation of LAMP1 and LAMP2 and the a2 isoform of V-ATPase (an enzyme involved in lysosomal acidification) are associated with increased activation of NF-kB and proinflammatory cytokines and chemokines in both uterus and placenta (Agrawal et al., 2015). Inhibition of autophagy can increase NF-kB activity and MMP-2 and MMP-2 expression levels in trophoblast cell lines (Oh et al., 2020). Activation of autophagy by rapamycin inhibits oxLDL-induced production of TNF-α and IL-6 in JEG-3 cells (Zhang et al., 2016). The expression of killer receptors including CD16, NKG2D, NKP30 and NKP46 in dNK cells was inhibited when co-cultured with rapamycin-pretreated HTR-8 trophoblasts, suggesting that autophagy activation may suppress inflammatory responses (Tan et al., 2020). In support of this, the administration of autophagy inhibitor, 3-MA promotes the cytotoxicity of uterine NK cells and increases the embryo absorption rate (Tan et al., 2020).

Our recent studies show that autophagy deficiency increases cell surface localization of GSDMD, and enhances the expression and colocalization of ASC and caspase 1 in extravillous trophoblasts exposed to sera from patients with severe PE not normal pregnancy (Cheng et al., 2019). This suggests that autophagy impairment can activate inflammasomes and promote inflammatory pyroptosis in stressed trophoblasts. In line with this, treatment of primary human trophoblasts with a lysosomal disruptor, chloroquine elevates the abundance of GSDMD both in the plasma and on the cell surface (Cheng et al., 2019). Our results suggest that impaired autophagy may contribute to sterile inflammation involved in PE pathology.

## Targeting Inflammasome and Autophagy for a Therapeutic Strategy for PE

### Targeting Inflammasome

The proper activation of the immune system is beneficial for the human body to combat inflammatory, degenerative, and metabolic diseases, but excessive inflammation may hyper-activate immune cells, which consequently switch on the NLRP3 inflammasome (Pirzada et al., 2020). The untimely activation of inflammasome as a consequence of placental ischemia needs critical understanding for developing targeted therapies for the prevention of PE pathology. Inhibition of the NLRP3 inflammasome or its downstream mediators may foster the development of novel anti-inflammatory therapies for the prevention or treatment of pregnancy complications. Currently, a variety of inhibitors are available that target activation of the NLRP3 inflammasome. Studies reported administration of silibinin (a flavonoid complex) could reduce NLRP3 activation upon MUS stimulated monocytes. Hooftman et al. (2020) reported that the kreb cycle-derived metabolite itaconate can attenuate NLRP3 Inflammasome activation, which could be evaluated for therapeutic intervention for PE, at least in the preclinical models of PE. In addition, NLRP3 inhibitor, MCC950, has been shown to prevent preterm birth and neonatal death in a mouse model of intra-amniotic inflammation (Faro et al., 2019; Gomez-Lopez et al., 2019b). In line with these observations, NLRP3 knock-out mice exhibit reduced the rates of preterm birth and neonatal death compared to wild-type mice in the presence of intra-amniotic inflammation (Motomura et al., 2020).

### Targeting Autophagy and Protein Aggregation

Targeting autophagy machinery has been proposed for developing therapeutic strategies to alleviate inflammation in a number of diseases such as neurodegenerative diseases (Ren and Zhang, 2018). Given the roles of autophagy in the suppression of inflammation, the degradation of protein aggregates, promotion of cell survival, improvement of mitochondrial quality control and removal of danger molecules such as cytosolic mitochondrial DNA, ROS and DAMPs, agents that enhance autophagy activity may confer multiple beneficial effects in addition to anti-inflammatory effect to treat the diseases. Whether increasing autophagy activity can alleviate the PE-related pathological alterations induced by protein aggregates, inflammation, ER stress, pyroptosis and other factors remain to be investigated.

## Author Contributions

SuS and SC conceptualized the work and contributed to writing and editing of the manuscript. All authors contributed to the article and approved the submitted version.

## Funding

This work was supported in part by the NIH P20 GM121298, 3P20GM121298-04W1 and P30 GM114750 grants, Brown University DEANS Award, Brown University Seed Award, and William and Mary Oh-William and Elsa Zopfi Professorship Award.

## Conflict of Interest

The authors declare that the research was conducted in the absence of any commercial or financial relationships that could be construed as a potential conflict of interest.

## Publisher’s Note

All claims expressed in this article are solely those of the authors and do not necessarily represent those of their affiliated organizations, or those of the publisher, the editors and the reviewers. Any product that may be evaluated in this article, or claim that may be made by its manufacturer, is not guaranteed or endorsed by the publisher.
